# Enclouage des os longs en situation précaire: intérêt du clou SIGN (*Surgical Implant Generation Network*)

**DOI:** 10.11604/pamj.2021.39.130.24190

**Published:** 2021-06-15

**Authors:** Pierre Girard, Moïse Cuma Zihindula, Delince Delinois, Lionel Kolontchang, Lewis Zirkle, Az-Eddine Djebara, Laurent Mathieu

**Affiliations:** 1 Service de Chirurgie Orthopédique et Traumatologique, Centre Hospitalier Universitaire Amiens-Picardie, Salouel, France,; 2 Service de Chirurgie Orthopédique et Traumatologique, Hôpital Universitaire de Kamenge, Bujumbura, Burundi,; 3 Service de Chirurgie Orthopédique et Traumatologique, Hôpital Universitaire de Mirebalais, Mirebalais, Haïti,; 4 Université de Yaoundé 1, Faculté de Médecine et de Science Biomédicale, Yaoundé, Cameroun,; 5 Surgical Implant Generation Network (SIGN), Richland, Wachington, United State of America,; 6 Service de Chirurgie Orthopédique, Traumatologique et Chirurgie Réparatrice des Membres, Hôpital d´Instruction des Armées Percy, Clamart, France

**Keywords:** Clou *Surgical Implant Generation Network*, enclouage centromédullaire, fractures des os longs, situation précaire, Surgical Implant Generation Network nail, centromedullary nailing, long bone fractures, precarious situation

## Abstract

Le développement économique des pays à faibles revenus a pour corollaire une augmentation considérable des véhicules motorisés, et en particulier des motocyclettes. Les accidents de la circulation augmentent ainsi que les fractures qui y sont associées. Le traitement des fractures des os longs repose dans la majorité des cas sur l´enclouage centro-médullaire verrouillé, procédé rarement disponible en situation sanitaire dégradée. Pour apporter à ces pays un traitement optimal, le clou SIGN (Surgical Implant Generation Network) a été développé en 1999 par Lewis Zirkle. Il est actuellement utilisé gratuitement dans 53 pays. En contrepartie une base de données internationale doit être remplie afin de l´évaluer et de le faire évoluer. A la lumière de nos expériences en Haïti et au Burundi et d´une revue de la littérature, nous décrivons ici ses particularités conceptuelles et techniques, dont l´implantation dans les pays francophones reste limitée.

## Abstract (en)

Economic development in low-income countries has led to a considerable increase in motor vehicles, in particular motorcycles. Traffic accident-related fractures are therefore increasing. The treatment of long bone fractures is, in the majority of cases, based on locked intramedullary nailing, a procedure which is rarely available in countries with poor sanitary conditions. To provide optimal treatment to these countries, the SIGN (Surgical Implant Generation Network) nail was developed in 1999 by Lewis Zirkle. It is currently used free of charge in 53 countries. In return, an international database must be completed in order to assess and develop it. In the light of our experiences in Haiti and Burundi and on the basis of a literature review, we here highlight the conceptual and technical features of SIGN nail whose implant in French-speaking countries is still limited.

## Perspective

**Introduction**: la progression économique qui accompagne le développement des pays à faible et moyen revenu a pour corollaire l´augmentation exponentielle des moyens de transports motorisés, notamment des motocyclettes. Le manque de moyens de protection combiné à la quasi-inexistence de prévention routière a pour effet d´augmenter la gravité des accidents de la route [1]. En parallèle, il existe une progression constante du nombre de traumatisés graves tenant à la migration des populations vers les villes. Les traumatismes font chaque année cinq millions de morts dans le monde, dont 25% par accident de la route. La mortalité traumatique dépasse celle liée aux infections par le VIH, la tuberculose et le paludisme combinées. Les pays émergeants sont pourvoyeurs de 90 % de ces accidents mortels. Vingt à 50 millions de personnes sont touchés par les accidents de la circulation dans le monde, la plupart avec des lésions sévères invalidantes [2].

Ces accidents à haute énergie cinétique entrainent fréquemment des fractures des os longs (humérus, fémur, tibia) dont le traitement est délicat en situation précaire [3]. Les hôpitaux régionaux disposant de moyens d´ostéosynthèse limités, la prise en charge n´est souvent pas optimale à la phase initiale. Cela aboutit à des complications secondaires (infection, non consolidation, cal vicieux) dont le traitement est encore plus problématique dans ce contexte. De plus, les patients et leur famille doivent assumer le coût du traitement chirurgical, qui reste souvent inaccessible aux populations les plus pauvres [3]. L´enclouage centromédullaire verrouillé est le traitement idéal des fractures du fémur et du tibia, et une option validée pour celui de la diaphyse humérale. Afin de rendre ces options thérapeutiques accessibles au plus grand nombre dans les pays émergents, Lewis G Zirkle a développé un système d´enclouage polyvalent dédié à ce contexte de soins: le clou SIGN (*Surgical Implant Generation Network*) [4]. A la lumière de notre expérience en Haïti et au Burundi, nous présentons ici les caractéristiques et intérêts de ce dispositif d´enclouage qui reste pour l´instant peu utilisé dans les pays francophones.

**Figure 1:**
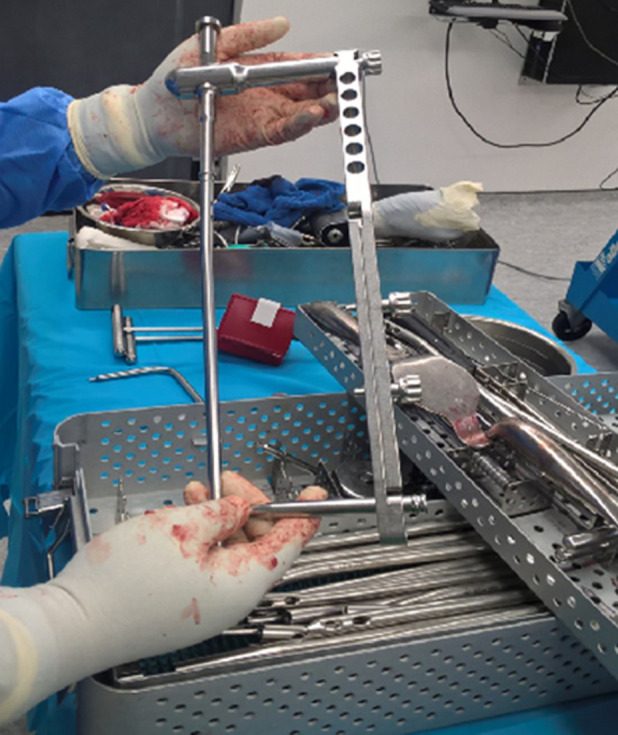
clou SIGN avec son ancillaire et son verrouillage distal (Haïti)

**Figure 2:**
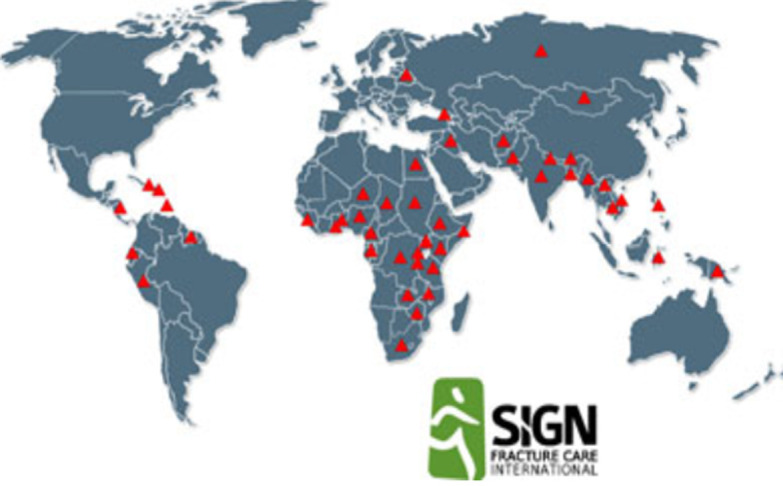
utilisation du clou SIGN à travers le monde

**Généralités, concept et indications du clou SIGN**: le clou SIGN est un clou centromédullaire développé en 1999 spécifiquement pour les pays en développement (Figure 1). Il est actuellement utilisé dans 359 hôpitaux au travers de 53 pays (Figure 2). Plus de 250.000 patients ont déjà bénéficié d´un traitement par ce clou [5-12]. Sa mise à disposition est gratuite pour les pays à faible revenu. Pour acquérir le clou SIGN, une demande doit être adressée à SIGN care international [13]. Deux conditions sont nécessaires à son acquisition: d´une part, un accompagnement par un chirurgien confirmé connaissant le clou SIGN pour la réalisation des premiers, d´autre part l´inclusion de tous les patients opérés dans la banque de données internationale SOSD (SIGN *Online Surgical Database*) en fournissant les radiographies préopératoires, postopératoires et celles réalisées lors du suivi ultérieur [6]. Cette base de données demeure à ce jour la plus importante dans les pays en voie de développement. Son objectif est d´analyser les mécanismes traumatiques à l´origine des fractures, ainsi que les modalités et les résultats de leur traitement. Le clou SIGN a quatre particularités essentielles qui le distinguent des systèmes d´enclouage conventionnels: 1) il peut être implanté sans amplificateur de brillance. En effet, la disponibilité dans les pays à bas et moyens revenus de ce dispositif radiologique est souvent inexistante. 2) Sa pose peut s´effectuer sans table orthopédique. Le corollaire à cela est parfois la nécessité d´ouverture du foyer de fracture afin de le réduire, notamment au niveau du fémur. 3) Il dispose d´un ancillaire de guidage pour le verrouillage distal sans recourir à la fluoroscopie per-opératoire spécifique (Figure 1). 4) Il s´agit d´un clou unique, disponible en diverses tailles, pour l´enclouage du fémur, du tibia et de l´humérus.

**Figure 3:**
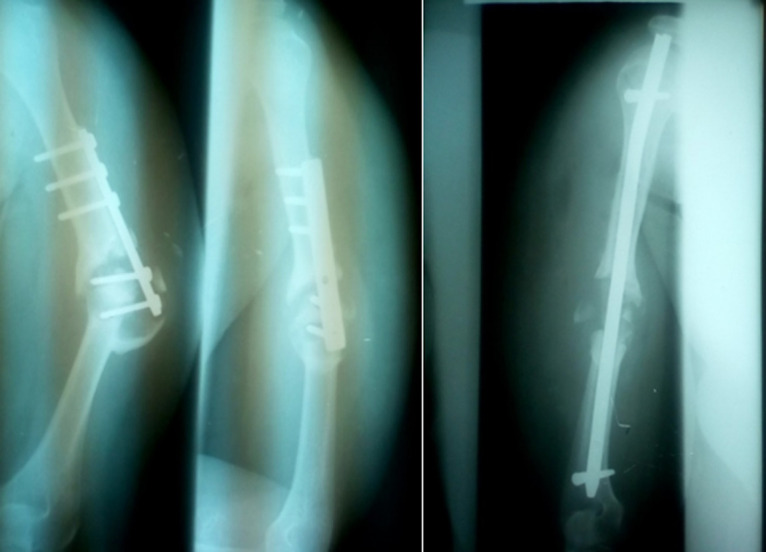
pseudarthrose de l´humérus après ostéosynthèse par plaque; traitement par greffe cortico-spongieuse et clou SIGN verrouillé standard (Bujumbura-Burundi)

Il s´agit d´un clou droit plein présentant une angulation proximale de 9°et une angulation distale de 1,5° disposant donc de 3 points de fixation osseux augmentant ainsi la stabilité. Le fait qu´il soit plein offre moins de surface pour la déposition d´un biofilm bactérien. Le clou standard a un diamètre variant de 8 à 12 mm, et une longueur 220 à 420 mm. Le nouveau clou Fin Nail est disponible quant à lui du diamètre 7 à 10 mm et de la longueur 240 à 320 mm. Il s´agit du même clou pour l´enclouage de l´humérus, du tibia, et du fémur en rétrograde ou antérograde mais sans besoin de verrouillage distal du fait de sa forme. En effet il dispose en distalité de crans venant s´encastrer dans le fût osseux, évitant ainsi la rotation. Le clou SIGN peut aussi être utilisé pour le traitement des pseudarthroses des os longs (Figure 3), qu´elles soient aseptiques ou septiques, ainsi que pour la réalisation d´arthrodèse de la cheville. Sur une série multicentrique de 250 patients présentant une pseudarthrose infectée du fémur et du tibia, Shah *et al*. [7] ont obtenu des résultats très encourageants avec un taux de 85% de consolidation après la 1ère intervention chirurgicale. Ce clou est également adapté au traitement des fractures des os longs en pédiatrie [8]. L´enclouage des fractures du fémur chez les préadolescents (dès 12 à 14 ans) est maintenant devenu la règle dans nos pays depuis quelques années. Face aux possibles écueils et complications rencontrés avec les enclouages élastiques stables, l´enclouage centromédullaire verrouillé lui est maintenant préféré dans cette tranche d´âge. Le clou SIGN standard ou sa variante le *Fin Nail* peuvent être utilisés pour le traitement des fractures du fémur chez cette population [8].

### Techniques de pose du clou SIGN

**Réduction**: la réduction peut se faire à foyer fermé essentiellement pour l´humérus et le tibia avec les techniques habituelles (davier, pointeau, *blocking screw* pour le tibia proximal...). Un abord même minime peut être nécessaire afin d´obtenir une bonne réduction, notamment au fémur. La réduction pour la pose du clou SIGN ne nécessite pas de table orthopédique. En revanche, une réduction à ciel ouvert des fractures est souvent nécessaire, notamment lorsque leur prise en charge est tardive. Un distracteur SIGN est disponible pour faciliter la réduction des fractures du fémur.

**Alésage**: le clou SIGN dispose d´alésoirs à main utilisables sans moteur. Ces alésoirs doivent être tournés dans le sens des aiguilles d´une montre durant tout l´alésage. Il peut se faire à foyer fermé ou à foyer ouvert si un abord de la fracture s´est imposé pour la réduction. Il se fait par augmentation progressive des incréments en commençant par le plus petit. En l´absence d´infection, le produit d´alésage est récupéré dans une cupule afin d´être remis dans le foyer de fracture. L´alésage est fait à la sensation tactile par le chirurgien et doit être supérieur de 2 mm au diamètre du clou utilisé. Enfin, les alésoirs sont gradués pour déterminer directement la longueur du clou à implanter.

**Implantation du clou**: le clou SIGN est monté sur le porte-clou et l´ancillaire de verrouillage distal avant son introduction dans le canal médullaire. Le verrouillage distal est effectué en premier à l´aide de l´ancillaire dédié. Il s´agit de l´étape la plus délicate de l´intervention puisqu´il s´agit d´aligner l´ancillaire et l´orifice de verrouillage distal du clou sans amplificateur de brillance. Des instruments spécifiques (solid slot Finder et curved slot Finder) permettent de faciliter cette étape. Une fois le verrouillage distal effectué, il alors possible d´effectuer une compression rétrograde du foyer de fracture. Le verrouillage proximal est effectué en dernier à l´aide du porte-clou. Il ne présente aucune spécificité : l´utilisation de solid slot Finder n´est ici pas nécessaire.

**Figure 4:**
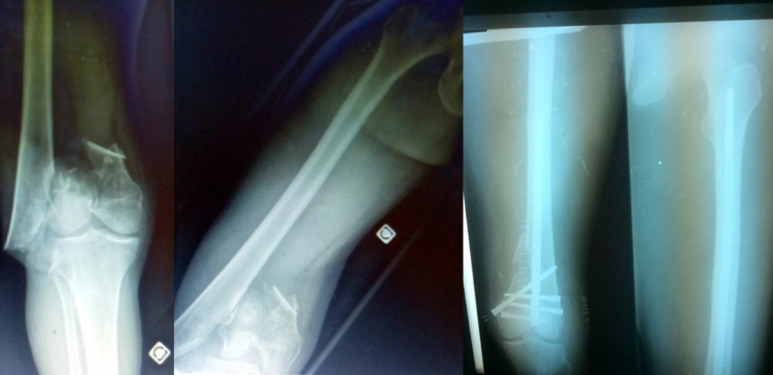
fracture sus et inter condylienne du fémur distal et radiographies post opératoires après implantation d´un *Fin Nail* (Bujumbura - Burundi)

**Spécificités au fémur**: l´enclouage du fémur peut se faire de façon antérograde ou rétrograde sur un patient en décubitus latéral. En l´absence de table orthopédique, la réduction se fait la plupart du temps à foyer ouvert. Les fractures du fémur distal peuvent être enclouées de façon rétrograde par une abord articulaire au niveau du genou (Figure 4). Le même clou pouvant être mis soit en rétrograde soit en antérograde. L´angulation du clou SIGN et son ancillaire permettent cette dualité [9-10]. Une extension du clou existe pour permettre le traitement des fractures trochantériennes.

**Spécificité au tibia et à l´humérus**: la mise en place du clou SIGN pour le tibia et l´humérus ne présente pas de particularités essentielles qui divergent des clous utilisés dans les pays occidentaux. Il faudra se reporter aux fiches très détaillées de la pose du clou SIGN [5]. La réduction au niveau du tibia et ses particularités ont été détaillées par Feibel et Zirkle [11].

***Fin Nail***: le clou *Fin Nail* (Figure 4) est une évolution du clou centromédullaire SIGN. Sa particularité réside dans le fait qu´iI permet un enclouage du fémur et de l´humérus sans vis de verrouillage distal. En effet il présente à sa partie distale un renflouement avec des stries ce qui lui permet de s´ancrer de manière stable et solide dans l´os. Ses indications sont les fractures stables de l´humérus et du fémur. Sa technique de pose est identique au clou SIGN standard sauf en ce qui concerne l´alésage. Sa mise en place nécessite au préalable un triple alésage [5] afin de permettre une fixation distale optimale dans l´os et d´éviter la rotation. Les résultats fonctionnels obtenus pour le clou Fin Nail sont très encourageants, notamment chez l´adolescent [8].

**Mise en compression du foyer et ablation du clou**: un dispositif peut être utilisé pour effectuer une compression rétrograde du foyer de fracture lors de l´implantation du clou afin de faciliter la consolidation. Il faut pour cela débuter par le verrouillage distal avant d´appliquer la compression rétrograde à l´aide d´une masselotte, puis d´effectuer le verrouillage proximal. Cet ancillaire d´extraction est obligatoirement utilisé pour le retrait du clou en cas de complications ou de gêne une fois que la consolidation est acquise.

**Complications et revue de la littérature**: la base de données SOSD permet une évaluation précise des performances du clou SIGN malgré la précarité du contexte sanitaire. Son analyse fournit les bases aux modifications et évolution futures du clou SIGN. Elle permet également une analyse des complications aigues et tardives de la mise en place du clou. Il a notamment été montré que les taux d´infection après pose du clou SIGN dans les pays en développement étaient comparables à ceux des pays occidentaux, en dépits du recours à la réduction à ciel ouvert [12,14]. Une revue de la littérature et une métanalyse a été effectuée en 2018 [15] référençant tous les articles sur PubMed/MEDLINE et la base de données Cochrane sur le clou SIGN et son utilisation pour les fractures du fémur, du tibia et de l´humérus. Les 14 études répertoriées retrouvaient un taux de suivi des patients de 23% avec un taux global de complications de 5,2 %. La plus courante était le cal vicieux, suivi par la pseudarthrose, l´infection puis la faillite du matériel. Les auteurs mentionnaient que ce taux de complications pouvait être en partie réduit en sélectionnant un diamètre de clou plus important et en utilisant plus de vis pour le verrouillage.

## Conclusion

Le clou SIGN apporte une solution efficace à la prise en charge des fractures des os longs dans les pays à faible ou moyen revenu. Sa mise à disposition gratuite permet de limiter le coût du traitement pour les patients et leur famille. Ce clou polyvalent permet le traitement des fractures du fémur, du tibia et de l´humérus avec un seul ancillaire sans amplificateur de brillance, ni table orthopédique. Une diffusion plus large de ce dispositif serait profitable dans nombres de pays en développement qui en sont encore dépourvus.

## References

[1] Richard Gosselin A, David Spiegel A, Richard Coughlin, Lewis Zirkle G (2009). Injuries: the neglected burden in developing countries. Bull World Health Organ.

[2] Gosselin RA (2009). The increasing burden of injuries in developing countries: direct and indirect consequences. Tech Orthop.

[3] Zirkle LG (2008). Injuries in developing countries: how can we help? the role of orthopaedic surgeons. Clin Orthop Relat Res.

[4] Haonga BT, Zirkle LG (2015). The SIGN Nail: factors in a Successful Device for Low-Resource Settings. J OrthopTrauma.

[5] Zirkle LG (2008). Technique Manual of SIGN IM Nail & Interlocking Screw System Insertion & Extraction Guide.

[6] Clough JF, Zirkle LG, Schmitt RJ (2010). The Role of SIGN in the Development of a Global Othopaedic Trauma Database. Clin Orthop Relat Res.

[7] Shah RK, Singh RP, Quassem F, Faruquee SR, Harison J (2009). SIGN Interlocking Nail for treatment of infected nonunion. Tech Orthop.

[8] Chen AR, Morris WZ, Zirkle LG, LIU RW (2018). Evaluation of intramedullary fixation for pediatric femoral shaft fractures in developing countries. J Orthop Trauma.

[9] Sekingi P, Okike K, Zirkle LG, Jawa A (2001). Femoral fracture fixation in developing countries: an evaluation of the Surgical Implant Generation Network (SIGN) intramedullary nail. J Bone Joint Surg Am.

[10] Carsen S, Park S, Simon DA, Feibel RJ (2015). Treatment with the SIGN nail in closed Diaphysal Femur fractures Results in Acceptable Radiographic Alignment. Clin Orthop Relat Res.

[11] Feibel RT, Zirkle LG (2009). Use of Interlocking Intramedullary Tibial Nails in Developing Countries. Tech Orthop.

[12] Young S, Lie SA, Halla G, Zirkle LG, Engesaeter LB, Havelin LI (2011). Low infection rates after 34,361 intramedullary nail operations in 55 low and middle income countries. Acta Orthopaedica.

[13] (2021). SIGN Fracture Care For surgeons. Mai.

[14] Young S, Lie SA, Halla G, Zirkle LG, Engesaeter LB, Havelin LI (2013). Risk factors for infection after 46,113 intramedullary nails operations in low and middle-income countries. World J Surg.

[15] Usoro AO, Bhashyam A, Mohamadi A, Dyer GS, Zirkle LG, Keudell AV (2019). Clinical outcomes and complications of the Surgery Implant Generation network (SIGN) intramedullary nail: a systematic review and meta-analysis. J Orthop Trauma.

